# Fatal Subacute Hepatic Failure in a Patient with AA-Type Amyloidosis: Case Report

**DOI:** 10.4061/2010/648089

**Published:** 2010-05-16

**Authors:** Ibrahim Altraif, Fayaz A. Handoo, Khaled O. Alsaad, Adel Gublan

**Affiliations:** ^1^Department of Hepatobiliary and Liver Transplantation, King Abdulaziz Medical City, P.O. Box 22490, Riyadh 11426, Saudi Arabia; ^2^Department of Pathology and Laboratory Medicine, College of Medicine, King Saud bin Abdulaziz University for Health Sciences and King Abdullah International Research Center, King Abdulaziz Medical City, P.O. Box 22490, Riyadh 11426, Saudi Arabia

## Abstract

Although systemic amyloidosis of amyloid-associated protein (AA) type (secondary or reactive amyloidosis) frequently involves the liver, it rarely causes clinically apparent liver disease. Mild elevation of alkaline phosphatase and hepatomegaly are the most common biochemical and clinical findings, respectively. We report a case of systemic amyloidosis of AA type, which clinically presented as subacute hepatic failure and resulted in a fatal clinical course in a 69-year-old man. To the best of our knowledge, this is the fifth case of hepatic amyloidosis of AA type that clinically presented as fatal subacute hepatic failure, an unusual clinical presentation for hepatic involvement by systemic AA-type amyloid.

## 1. Introduction

Hepatic involvement in systemic amyloidosis is common and occurs in myeloma-related (AL) (primary amyloidosis) and amyloid-associated (AA) (secondary or reactive) types of the disease. However significant clinical evidence of hepatic dysfunction is not common and the patients rarely exhibit clinical manifestations. These may include hepatomegaly, mild jaundice, and, rarely, severe cholestasis. Portal hypertension may complicate hepatic amyloidosis, and subcapsular hematoma and spontaneous rupture of liver were reported [1]. Subacute and acute, fulminant liver failures were previously reported, particularly in association with AL-type amyloidosis in the setting of myeloma [2]. Herein, we present an unusual case of AA-type amyloidosis with massive hepatic involvement and subacute liver failure—a clinical presentation rarely seen in secondary hepatic amyloidosis.

## 2. Case Report

A previously healthy 69-year-old man presented with history of progressively increasing jaundice for six weeks and abdominal distension with swelling of the feet for four weeks. He also had symptoms of fatigue, poor appetite, and mild generalized itching. His past medical history was unremarkable, with no previous history of chronic liver disease. Upon physical examination, the patient had normal built exhibited severe jaundice, bilateral pitting lower-limb edema, left-sided moderate pleural effusion, short apical systolic murmur, and massive hepatomegaly, associated also with marked ascites without signs of overt congestive heart failure. Complete blood count and serum electrolytes were within normal limits. Laboratory studies showed blood urea 3.9 mmol/l (normal range 3–9.2), creatinine 64  *μ*mol/l (45–110), total bilirubin of 196 *μ*mol/l (3.4–20.5), serum albumin 24 g/l (35–50), INR 3.2, alkaline phosphatase 256 IU/l (40–150), *γ*-glutamyltransferase 136 (2–30), alanine aminotransferase 28 IU/l (5–55), and aspartate aminotransferase 70 IU/l (5–34). The C-reactive protein was elevated, and immunoglobulin levels were normal with immunoglobulin (Ig) G 12 g/l (7.51–15.60), IgA 3.15 g/l (0.82–4.53), and IgM 0.52 g/l (0.46–3.04). Serum protein electrophoresis was normal and with negative urine measurement of Bence-Jones protein. Alpha-fetoprotein was within normal range. Viral serology for HCV was negative, while HBcAb and HBs-Ab were positive. Rheumatoid factor, antinuclear antibody, antismooth muscle antibody, and anti-DNA antibody were all negative. Twenty-four-hour urinary protein excretion was 0.068 g/l. Abdominal ultrasonography revealed hepatomegaly with no focal liver lesions and normal intra- and extrahepatic bile ducts. The size of the spleen was normal, and the inferior vena cava as well as hepatic, portal, and superior mesenteric veins were patent. Transthoracic echocardiography showed severe concentric left ventricular hypertrophy with diastolic dysfunction, normal systolic function with ejection fraction of >55%, a marginally elevated right ventricular systolic pressure at 30–40 mmHg, and a small pericardial effusion. Ascetic tap was performed, revealing normal white cell-count and total protein of 7.6 g/l. Transjugular liver biopsy was performed. 

**Figure 1:**
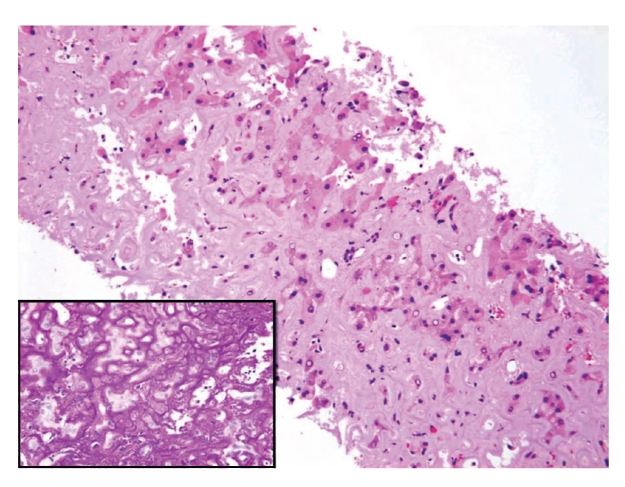
Liver tissue with massive deposition of extracellular pale eosinophilic amyloid material (H&E, × 200). The amyloid deposition exhibits predominantly a sinusoidal pattern (Inset, Periodic Acid Schiff stain, × 400).

**Figure 2:**
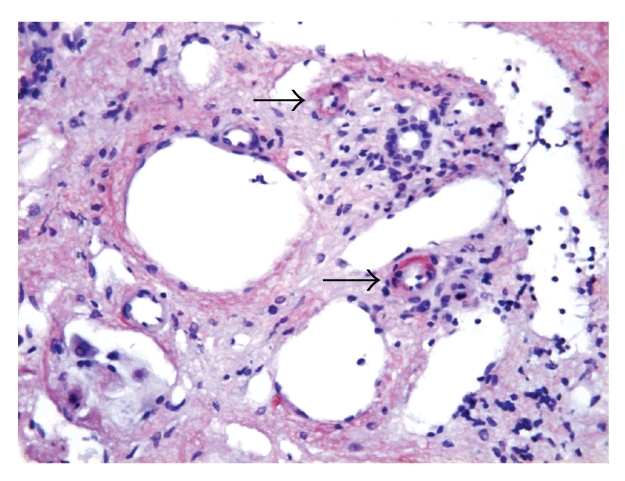
The amyloid material is stained orange-red by Congo Red stain. Focal deposition of amyloid was identified in the arterioles in the portal tracts (arrows) (× 400).

**Figure 3:**
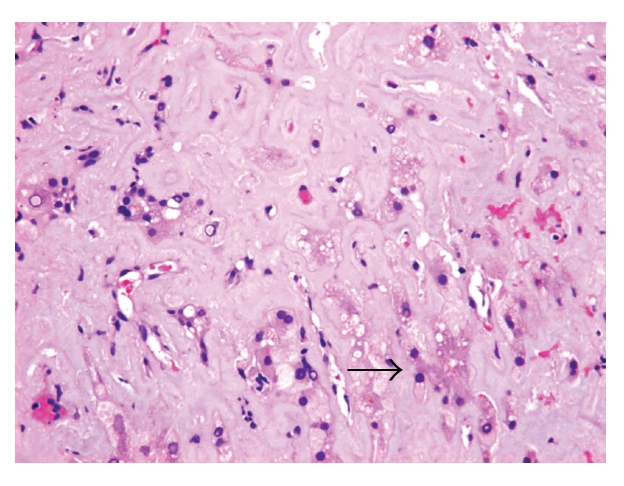
Scattered atrophic hepatocyte plates present within the massive amount of amyloid material (arrow) (H&E, × 400).

**Figure 4:**
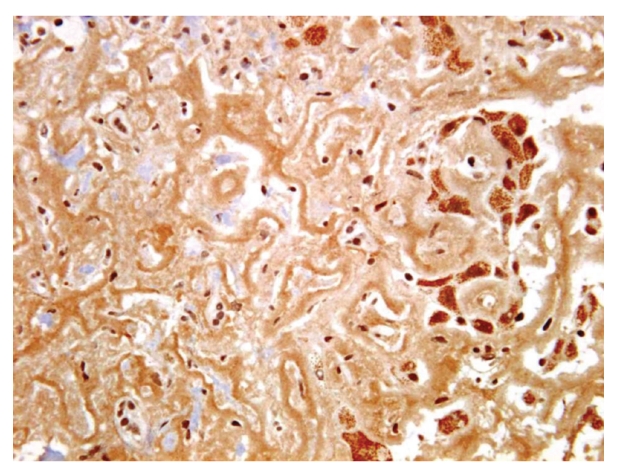
Positive immunohistochemical staining for amyloid-associated protein, highlighting the sinusoidal linear deposition of amyloid (× 400).

Five-micron sections from the 10% formalin-fixed and paraffin-embedded tissue were examined. Histological examination was performed on haematoxylin and eosin, periodic acid Schiff with and without diastase pretreatment, Masson Trichome, van Gieson, and reticulin special stains. Persian blue and rhodanine special stains were also performed to evaluate the hepatic iron and copper contents, respectively. Congo Red special stain for amyloid was performed on 8-micron sections. Light microscopy revealed liver tissue with diffuse, panacinar effacement of the hepatic architecture by extensive, pale eosinophilic, homogenous, and acellular material, causing severe loss of liver parenchyma. This material was predominantly deposited perisinusoidally in linear pattern (sinusoidal pattern) (Figure 1). Focally, deposition of the material was also identified in the interlobular arterioles (Figure 2). The material was stained positive with Congo Red and displayed green birefringence when viewed under polarized light, confirming amyloid deposition. Scattered residual, atrophic hepatocytes and bile ducts were present (Figure 3). Focal mild lymphocytic chronic portal inflammation was noted. Two additional ancillary studies were performed: (1) immunohistochemical staining for AA-type amyloid (Dilution 1 : 100, clone mc1, Dako, Denmark), which was diffusely positive (Figure 4), (2) electron microscopy examination using formalin-fixed and paraffin-embedded tissue, which revealed randomly distributed nonbranching rigid fibrillary structures of 7 to 10 nm in diameter ultrastructural features compatible with amyloid. 

The patient's clinical condition rapidly deteriorated over the subsequent two weeks, and he developed fulminant liver failure with marked elevation of the total bilirubin (645 *μ*mol/l), INR (4.4), and prothrombin 45.2 (NR 7.2–10.4 sec) as well as partial thromboplastin 72.5 (NR 26.1–37.3 sec) times. These were associated with the development of acute renal failure, the blood urea was 13.1 mmol/l, and creatinine was 141 *μ*mol/l. The patient's condition continued to deteriorate further and he expired on day 54 of his initial presentation.

## 3. Discussion

Amyloidosis is a medical condition of abnormal protein metabolism, characterized by extracellular deposition of misfolded, normally soluble proteins and polypeptides in fibrillary form. Amyloidosis is classified on the basis of the chemical composition of the amyloid fibrils and their precursor protein to morphologically identical but chemically different types. The two principle types of amyloidosis are (1) myeloma-associated (AL) (primary) amyloidosis, which is associated with plasma cell dyscrasias and malignant B-cell-type lymphoproliferative malignancies and characterized by the deposition of the variable region of the immunoglobulin kappa or lambda light chains and (2) amyloid-associated (AA) (secondary or reactive) amyloidosis, which is associated with chronic infectious and noninfectious inflammatory conditions, Hodgkin lymphoma, and non-lymphoid malignancies and characterized by the deposition of amyloid A fibrils, which are derived from the serum AA precursor protein. Both types can be localized or systemic. The liver is commonly involved by systemic amyloidosis. Usually, hepatic amyloidosis is not associated with significant liver dysfunction and the most common biochemical and clinical presentations of hepatic amyloidosis are elevation in alkaline phosphatase levels and hepatomegaly, respectively [3]. Hepatic failure is not a common consequence of hepatic amyloidosis. Rare but well-documented cases of fatal subacute and acute fulminant hepatic failures were previously reported in associated with myloma-assocaited (AL) amyloidosis [2, 4, 5]. However, hepatic failure is exceedingly rare in the setting of liver involvement with amyloid-associated (AA) amyloidosis. To the best of our knowledge, only four cases of hepatic AA amyloidosis resulting in liver failure were reported in the English literature [6–9]. In the present case, the patient had no history of chronic inflammatory conditions or malignancy. He had no family history of amyloidosis. The clinical presentation of subacute liver failure with rapid deterioration in clinical condition and subsequent fatality is unusual for hepatic involvement by AA amyloidosis. Hepatic amyloid deposition in systemic AA amyloidosis is typically associated with significant amyloid deposition in other organs, and carries a poor prognosis. In a study by Lovat et al. [10], one hundred and thirty eight patients with AA amyloidosis were studied, in which 25 (18%) had hepatic involvement; in that study, there was significant drop in the five-year survival from 72% in patients without liver involvement to 43% in patients with liver involvement. The presences of congestive heart failure, high bilirubin level, and high platelet count have been found to be predictors of poor prognosis in these patients [9]. Our patient had two of the three poor prognostic factors; the high bilirubin and cardiac involvement. Although the patient was positive for HBc-Ab, he had high titers of HBsAb denoting immunity. Amyloidosis is rarely being considered in the differential diagnosis of acute or fulminant liver failure. In fact, in a study of 98 patients, amyloidosis was considered in the differential diagnosis only in 14 (14%) patients before liver biopsy [9]. High CRP and a low serum albumin level have been shown to be indicators of hepatic involvement in amyloidosis [6]. 

The amyloid characteristically appears acellular pale eosinophilic and amorphous on routine hematoxylin and eosin stain, and, when stained with Congo Red, it exhibits green birefringence under polarization. Different histological patterns of distribution were described in hepatic amyloidosis. These include vascular pattern, in which mainly the hepatic arteries and arterioles are involved, sinusoidal/linear pattern in which the amyloid deposits in the space of Disse along the hepatic sinusoids, and globular pattern that is characterized by the presence of 1–40 *μ*m round globules of amyloid in portal tracts, space of Disse, and occasionally in the cytoplasm of the hepatocytes [11]. These patterns of hepatic amyloid deposition occur singly or in conjunction and cannot be used to distinguish between the various forms of systemic amyloidosis. In a recent study Makhlouf and Goodman [12] suggested that the globular form of amyloidosis represents an early stage of hepatic involvement by systemic amyloidosis. 

In conclusion, this case describes a patient with systemic AA-type amyloidosis, who presented with fatal subacute liver, an unusual clinical presentation of the secondary hepatic amyloidosis. Hepatic amyloidosis should always be considered in the differential diagnosis of acute liver failure especially in patients with features suggestive of infiltrating disorder, such as massive hepatomegaly, intrahepatic cholestasis, unusually low serum albumin, and evidence of cardiac or other organs being involved. Liver biopsy to confirm the diagnosis may be warranted.
